# Epigenetic manipulation to improve mouse SCNT embryonic development

**DOI:** 10.3389/fgene.2022.932867

**Published:** 2022-08-30

**Authors:** Yamei Li, Qiang Sun

**Affiliations:** ^1^ University of Chinese Academy of Sciences, Beijing, China; ^2^ Institute of Neuroscience, CAS Key Laboratory of Primate Neurobiology, State Key Laboratory of Neuroscience, Center for Excellence in Brain Science and Intelligence Technology, Chinese Academy of Sciences, Shanghai, China; ^3^ Shanghai Center for Brain Science and Brain-Inspired Intelligence Technology, Shanghai, China

**Keywords:** somatic cell nuclear transfer, cloning efficiency, epigenetic barriers, pre-implantation, post-implantation

## Abstract

Cloned mammals can be achieved through somatic cell nuclear transfer (SCNT), which involves reprogramming of differentiated somatic cells into a totipotent state. However, low cloning efficiency hampers its application severely. Cloned embryos have the same DNA as donor somatic cells. Therefore, incomplete epigenetic reprogramming accounts for low development of cloned embryos. In this review, we describe recent epigenetic barriers in SCNT embryos and strategies to correct these epigenetic defects and avoid the occurrence of abnormalities in cloned animals.

## Introduction

Dolly, the first cloned mammal produced by SCNT, was born in 1996 (Wilmut et al., 1997). Since then, about 25 mammalian species have been cloned by SCNT (Wakayama et al., 1998; Lanza et al., 2000; McCreath et al., 2000; Onishi et al., 2000; Loi et al., 2001; Chesné et al., 2002; Forsberg et al., 2002; Keefer et al., 2002; Loi et al., 2002; Shin et al., 2002; Galli et al., 2003; Woods et al., 2003; Zhou et al., 2003; Gómez et al., 2004; Lee et al., 2005a; Lee et al., 2005b; Lagutina et al., 2005; Lan et al., 2006; Li et al., 2006; Berg et al., 2007; Green et al., 2007; Kim et al., 2007; Shi et al., 2007; Gómez et al., 2008; Folch et al., 2009; Hoshino et al., 2009; Wani et al., 2010; Yang et al., 2010; Hwang et al., 2013; Wani et al., 2017; Lu et al., 2018; Gavin et al., 2020), including our successful cloning of non-human primates (Liu et al., 2018b; Liu et al., 2019). Indeed, SCNT has great potential applications in many fields, such as agro-biotechnology, endangered species conservation, disease model production, and regenerative medicine (Kim et al., 2007; Rogers et al., 2008; Gómez et al., 2009; Hickey et al., 2011; Chung et al., 2014; Lu et al., 2014; Yamada et al., 2014; Chung et al., 2015; Lu et al., 2015). However, low efficiency hampers the application of SCNT and its extension. The standard SCNT efficiency is only 1%–5%, which results in a general failure of the technology to be applied in mammals extensively (Yang et al., 2007b; Loi et al., 2016; Matoba and Zhang, 2018). Somatic cells maintain some of their specific epigenetic memory, including DNA methylation, histone modification, chromosome configuration, and non-coding RNA expression, even though the oocyte has a powerful ability to reprogram (Dean et al., 2001; Mann et al., 2003; Santos et al., 2003; Yamagata et al., 2007; Zhang et al., 2009; Smith et al., 2012; Gao et al., 2018b; Matoba et al., 2018; Zhang et al., 2020). The efficiency of SCNT-based cloning in mammals remains at relatively or extremely low levels, and it appears to differ in a species-specific manner. To improve the SCNT efficiency, extensive efforts are needed to precisely identify the epigenetic factors and molecular mechanisms involved in the reprogramming of the nuclear donor cell’s (NDC’s) genome in somatic cell cloned embryos (Cao et al., 2020; Liu et al., 2020; Feng et al., 2022; Li et al., 2022). Thus far, the provenance and type of NDCs have been shown to influence the capability of donor genomic DNA to be epigenetically reprogrammed in the oocyte cytoplasm during SCNT (Samiec and Skrzyszowska, 2010, 2013; Sadeesh et al., 2016; Wiater et al., 2021b; Xu et al., 2021; Zhang et al., 2022). Moreover, the ability to reprogram the transcriptional activity of the donor cell genome in cloned embryos can be increased by extrinsic epigenetic modifiers (Sangalli et al., 2014; Sharma et al., 2018; Taweechaipaisankul et al., 2019; Skrzyszowska and Samiec, 2020; Wiater et al., 2021a). The approaches used to epigenomically modulate somatic cell nuclei in SCNT embryos have been thoroughly described in the review article by Samiec and Skrzyszowska (2018). Therefore, overcoming these epigenetic barriers and changing the somatic epigenome into an embryonic epigenome can allow for increased rates of full-term cloned embryo development.

**FIGURE 1:**
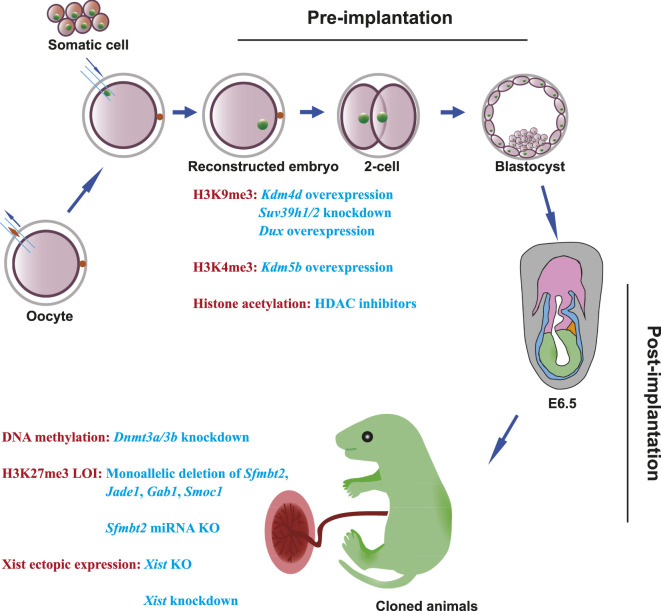
Epigenetic barriers and strategies to overcome developmental defects in SCNT embryos. During the development of SCNT embryos, epigenetic barriers can be classified into two categories: one is pre-implantation defects; the other is post-implantation defects. In pre-implantation of SCNT embryos, H3K9me3 deposition can be overcome by Kdm4 overexpression, *Suv39h1/2* knockdown, and *Dux* transient overexpression. H3K4me3 deposition can be solved by *Kdm5b* injection. HDAC inhibitors can alleviate histone acetylation. In post-implantation of SCNT embryos, *Dnmt3a/3b* knockdown can improve post-implantation development. To overcome the loss of imprinting in H3K27me3 imprinted genes, generation of monoallelic deletions of *Sfmbt2, Jade1*, *Gab1*, and *Smoc1*, or deletion of *Sfmbt2* miRNA can fix aberrant placentas and improve post-implantation development. Knockout or knockdown of *Xist* can solve the ectopic expression of Xist in cloned embryos.

Cloned mammals often have both placental and fetal abnormalities, which are derived from trophectoderm (TE) cells and the inner cell mass (ICM), respectively. For example, enlargement of the spongiotrophoblast (ST) layer, an irregular spongiotrophoblast–labyrinthine (LB) cell boundary, and proliferation of glycogen-rich cells in the placenta, as well as overweight conditions, respiratory disorders, and neonatal deaths have been observed in previous studies (Tanaka et al., 2001; Cibelli et al., 2002; Miglino et al., 2007; Palmieri et al., 2008; Matoba and Zhang, 2018). Fortunately, these abnormalities in cloned mammals cannot be transmitted to the offspring (Shimozawa et al., 2002; van der Berg et al., 2019; Shi et al., 2020). The two main barriers during SCNT embryo development are defective pre-implantation, which is the result of the failure of zygotic genome activation (ZGA) and embryonic arrest, and defective post-implantation development, which results in arrested gestation (Dean et al., 2001; Mann et al., 2003; Kishigami et al., 2006; Rybouchkin et al., 2006; Wang et al., 2007; Matoba et al., 2014; Gao et al., 2018b; Matoba et al., 2018; Yang et al., 2021). In this review, we describe and discuss the recent epigenetic barriers and strategies to improve pre-implantation and post-implantation development in cloned embryos and explore other strategies that can be implemented to overcome these barriers in mammals with a special focus on mice, owing to the many breakthroughs of SCNT in the mouse model (Figure 1).

## Epigenetic defects during pre-implantation development in cloned embryos

### Pre-implantation development in cloned embryos

Pre-implantation refers to the time during the zygote to blastocyst stages before implantation. There are two important events that occur during pre-implantation. One is the ZGA, and the other is the establishment of TE and ICM lineages (Braude et al., 1988; Mottla et al., 1995; Hamatani et al., 2004; Marikawa and Alarcón, 2009; Lee et al., 2014; Lu et al., 2016; Gao et al., 2018a; Schulz and Harrison, 2019). After fertilization, the genome is transcriptionally quiescent and is reprogrammed depending on maternal products. In mice, following fertilization, maternal RNA and protein are degraded, and the zygotic genome activates at the end of the 2-cell stage, wherein the maternal products are replaced by zygotic mRNA and protein. This process is known as ZGA (Lee et al., 2014; Jukam et al., 2017; Schulz and Harrison, 2019). Reconstructed cloned embryos also undergo ZGA since oocyte cytoplasm can initiate reprogramming when the somatic cell genome is introduced (Deng et al., 2020; Fu et al., 2021). Although this reprogramming can result in cloned animals successfully, the insufficient reprogramming by oocyte cytoplasm leads to low efficiency, and many SCNT embryos arrest at the ZGA stage (Matoba et al., 2014; Loi et al., 2016; Zhang et al., 2018c; Deng et al., 2018). The first two lineages emerge at the blastocyst stage: one is the ICM, which will give rise to the fetus, and the other is the TE, which will form the placenta. There are different epigenomes and transcriptomes between the fertilized blastocysts and SCNT blastocysts (Yang et al., 2007a; Zhao et al., 2010b; Marcho et al., 2015; Gao et al., 2018b). For example, many development-related genes are downregulated and affected by DNA methylation in SCNT blastocysts compared with the fertilized blastocysts (Gao et al., 2018b). Surprisingly, the transcriptomes are identical when comparing fertilized blastocysts to SCNT blastocysts after epigenetic modification, except that some H3K27me3-dependent imprinted genes are significantly upregulated in SCNT blastocysts (Matoba et al., 2018). These results indicate that additional epigenetic barriers remain in somatic cells, which prevent many SCNT embryos from going through ZGA and developing.

### H3K9me3 histone modification hampers proper zygotic genome activation

H3K9me3, a repressive histone modification, is associated with heterochromatin, and its deposition prevents transcription factor binding. Thus, H3K9me3 can silence genes and restrict developmental potency in early embryos (Becker et al., 2016). In 2014, *Matoba et al.* identified 222 genomic regions in cloned mouse embryos by comparative transcriptomic and epigenetic analysis. These genomic regions were termed reprogramming-resistant regions (RRRs) because they were aberrantly expressed in the mouse SCNT embryos at the late 2-cell stage when the major ZGA occurs. Furthermore, they found that the H3K9me3 modification was enriched in donor cell RRRs and served as a barrier to the initiation of ZGA and the activation of developmentally important genes in SCNT embryos (Matoba et al., 2014). Recently, Chen et al.(2020) found that the H3K9me3 barrier led to stronger topologically associating domains (TADs) and aberrant super-enhancer and promoter interactions in mouse SCNT embryos. Significantly, the H3K9me3 barrier was conserved in other mammalian SCNT embryos, including bovine, pig, human, monkey, and sheep (Cao et al., 2015; Chung et al., 2015; Huang et al., 2016; Liu et al., 2018a; Zhang et al., 2018a; Liu et al., 2018b; Zhang et al., 2018c; An et al., 2019). In mice, overcoming the somatic cell H3K9me3 barrier enables proper ZGA, and 88.6% of reconstructed embryos developed to the blastocyst stage, and 7.6% of transferred SCNT embryos developed to term (Matoba et al., 2014).

### H3K4me3 histone modification may activate inappropriate genes after somatic cell nuclear transfer

H3K4me3 is often regarded as an active histone modification, which promotes the expression of genes during embryo development. In 2016, *Liu et al.* reported that injection of *Kdm5b*, an H3K4me3 demethylase, rescued 4-cell arrest and achieved high-quality blastocysts after SCNT in mice (Liu et al., 2016). This result suggests that H3K4me3 is an epigenetic barrier for SCNT-mediated reprogramming, and activating inappropriate genes with ON-memory from donor somatic cells can lead to developmental abnormalities or arrest (Liu et al., 2016; Hörmanseder et al., 2017). Aside from mice, the H3K4me3 barrier from donor somatic cells also exists in *Xenopus*, goats, and bovines and impedes the development of SCNT embryos (Hörmanseder et al., 2017; Zhang et al., 2018c; Deng et al., 2020; Zhou et al., 2020). However, based on transcriptome and epigenome analysis, H3K4me3 modification is not accounted for the failure of ZGA in pigs (Liu et al., 2021). This result indicates that H3K4me3 as an epigenetic barrier may not be conserved in all mammals.

### Histone acetylation affects the development of somatic cell nuclear transfer embryos

Histone acetylation, which is the acetylation of lysines in histone tails, is associated with the active expression of genes. Acetylation of lysines can loosen chromatin and promote transcription (Clayton et al., 2006). Histone acetyltransferase (HAT) and histone deacetylase (HDAC) regulate the landscape of acetylation, and the sites of acetylation include highly conserved regions in histones H4 (K5, K8, K12, and K16) and H3 (K9, K14, K18, K23, and K27) (Kurdistani et al., 2004). Previous studies of reprogramming during SCNT demonstrated that H3K9ac, H3K14ac, and H4K16ac can be quickly deacetylated in donor somatic cells and acetylated after activation. However, H4K8ac and H4K12ac still maintain the acetylation pattern that resembles donor somatic cells (Wang et al., 2007). Moreover, in 2021, *Yang et al.* identified aberrant regions of H3K9ac in SCNT embryos that were associated with many important genes for embryonic development. Furthermore, these aberrant H3K9ac regions led to the failure of ZGA (Yang et al., 2021). Thus, H3K9ac may be an epigenetic barrier to proper ZGA. Notably, the barrier of histone acetylation in preimplantation of SCNT is conserved in large mammals because inhibitors of histone deacetylase (HDACi) can promote blastocyst development (Akagi et al., 2011; Hou et al., 2014; Wen et al., 2014; Min et al., 2016; Jin et al., 2017; Wang et al., 2018) but lead to no significant increase in birth rates (Sangalli et al., 2012; Hosseini et al., 2016; Loi et al., 2016; Ogura et al., 2021).

### DNA methylation may act as a barrier to the development of somatic cell nuclear transfer embryos

DNA methylation is an abundant and vital epigenetic modification in development and is associated with gene silencing. 5-Methylcytosine (5mC) exists in the mammalian genome in 60%–80% of CpG sites (Smith and Meissner, 2013; Wu and Zhang, 2017). The DNA methyltransferase (DNMT) family is responsible for the generation of 5mC and is classified into two categories: *de novo* methyltransferase and maintenance methyltransferase. *De novo* methyltransferases (DNMT3A and DNMT3B during the development of embryos) are responsible for initial methylation (Okano et al., 1999; Wu and Zhang, 2017). DNMT1, which methylates hemi-methylation DNA through UHRF1, can maintain 5mC through cell divisions during development (Hermann et al., 2004; Bostick et al., 2007; Sharif et al., 2007; Wu and Zhang, 2017). Both *de novo* and maintenance methyltransferases are crucial because deficiencies of Dnmt3b or Dnmt1 result in fetuses that look normal before E9.5 but die at the later developmental stage in the mouse, and Dnmt3a-null mice die at 4 weeks of age (Li et al., 1992; Okano et al., 1999). It is crucial to regulate DNA methylation dynamically during development. There are two ways to mediate DNA demethylation. One is passive DNA demethylation, which occurs through dilution of 5mC following replication, and the other is active DNA demethylation by the TDG–TET pathway (Wu and Zhang, 2010; Wu and Zhang, 2014; Bochtler et al., 2017).

In mammalian pre-implantation development, DNA demethylation and re-methylation both occur and play a crucial role during this process. After fertilization, the oocyte and sperm lose their DNA methylation pattern except in imprinted regions, and the paternal genome is initially demethylated by the maternally stored Tet3 proteins (Greenberg and Bourc’his, 2019; Gu et al., 2011; Iqbal et al., 2011; Wossidlo et al., 2011). The maternal genome also undergoes demethylation, but much less than the paternal genome (Shen et al., 2014). Then, passive demethylation takes place in two parental genomes through DNA replication-dependent dilution (Howell et al., 2001). At the blastocyst stage, DNA methylation is at its lowest and is followed by DNA re-methylation by DNMT3A and DNMT3B after implantation (Zhang et al., 2018b; Zhu et al., 2018).

DNA methylation erasure and establishment also take place in SCNT embryos. Like after fertilization in mice, the pseudopronucleus (PPN) in cloned embryos undergoes active demethylation through TET3 action stored in the oocyte (Wossidlo et al., 2010; Gu et al., 2011; Wossidlo et al., 2011; Matoba and Zhang, 2018). In the first division stage, the high methylation level of the SCNT embryo is still similar to that in the donor somatic cell, and reprogramming of DNA methylation is not complete. After several rounds of replication dilutes the DNA methylation, the global methylation patterns of SCNT blastocysts after epigenetic modification are similar to those of IVF blastocysts (Ma et al., 2014; Matoba et al., 2018). This result indicates that general DNA methylation is reprogrammed in SCNT embryos successfully. However, it has been shown that *Magea* and *Xlr* clusters of the X chromosomes still failed to be activated and maintain high levels of promoter DNA methylation in most of these genes, which suggests DNA methylation may act as a barrier to reprogramming (Matoba and Zhang, 2018). In 2018, *Gao et al.* found there were re-methylated regions enriched in LTR elements in mouse cloned embryos during the 2-cell and 4-cell stages, and these re-methylated regions resulted in improper expression of genes and retrotransposons that are important for ZGA (Gao et al., 2018b). In addition to mice, other mammals also have abnormal DNA methylation patterns during pre-implantation as revealed by 5mC antibody-based immunostaining, bisulfite sequencing, WGBS, and MeDIP-sequencing (Dean et al., 2001; Beaujean et al., 2004; Li et al., 2014; Zhang et al., 2016; Song et al., 2017; Wang et al., 2020b; Deng et al., 2020; Malpotra et al., 2022). From the aforementioned SCNT studies, overexpression of *Kdm4d* and *Xist* KO may also help SCNT embryos through the DNA methylation barrier. However, more evidence is needed to prove the DNA methylation barrier from donor somatic cells and improve cloning efficiency after modifying DNA methylation.

## Epigenetic barriers after implantation in cloned embryos

### Post-implantation development in cloned embryos

After the SCNT blastocyst implants into the uterus, TE cells undergo proliferation and form extraembryonic tissues including the placenta, and ICM cells develop into the embryo proper and yolk sac tissue (Arnold and Robertson, 2009; Rossant and Tam, 2009). During this process, not only the morphology of the embryo changes dramatically, but the epigenome of the post-implantation embryo also goes through reprogramming. For example, DNA methylation is re-established around implantation in mouse embryos. The loss of non-promoter (distal) H3K27me3 is seen after implantation. Moreover, H3K27me3 maternal imprinting was replaced with DNA methylation in extraembryonic tissues, and H3K27me3 maternal imprinting was missing after implantation (Wu and Zhang, 2010; Zheng et al., 2016; Chen et al., 2019; Xia and Xie, 2020).

### DNA methylation may be an epigenetic barrier for somatic cell nuclear transfer post-implantation development

In post-implantation fertilized embryos, DNA hypomethylation in extraembryonic tissues can be sustained until birth, and the epiblast genome gains DNA methylation soon after implantation (Zhang et al., 2018b). However, due to a lack of critical data about DNA methylation in SCNT embryos post-implantation, we cannot compare the DNA methylation between fertilized and SCNT post-implantation embryos. Nevertheless, *Gao et al.* found the combination of DNA and histone modifier treatments not only enhanced the poor cloning efficiency but also alleviated abnormal SCNT placental development. It is worth mentioning that histone H3K9me3 modification could not rescue placental abnormalities. Thus, DNA methylation may be an epigenetic barrier for SCNT post-implantation (Gao et al., 2018b), and more evidence is needed to verify this conclusion.

### Loss of H3K27me3-dependent imprinting in somatic cells impedes somatic cell nuclear transfer post-implantation development

It is well known that abnormal placentas are often seen in mammalian SCNT embryos. Recent studies showed that loss of H3K27me3-dependent imprinted genes leads to abnormal placentas (Wang et al., 2020a; Inoue et al., 2020; Xie et al., 2022). H3K27me3, similar to DNA methylation, is a repressive modification that can repress gene expression. In mice, H3K27me3 modifications from sperm are erased globally upon fertilization, and only non-promoter (distal) H3K27me3 modifications from the oocyte are maintained. Moreover, the erasure of promoter H3K27me3 upon fertilization was reestablished extensively in developmental genes in post-implantation embryos (Zheng et al., 2016).

Okae et al. (2014) found the loss of imprinting of *Gab1*, *Sfmbt2*, and *Slc38a4* in cloned mice, including in the brain and placenta through transcriptome-wide analyses. Furthermore, they found that *Gab1*, *Sfmbt2*, and *Slc38a4* were maternally repressed independent of DNA methylation in oocytes for the establishment of imprinting (Okae et al., 2014). These results suggest there exists a DNA methylation-independent imprinting mechanism to account for the loss of imprinting of *Gab1*, *Sfmbt2*, and *Slc38a4.* In 2017, Inoue *et al.* used a low-input DNase I-sequencing (liDNase-seq) technique and found a DNA methylation-independent mechanism that prevents accessibility of certain maternal alleles in the zygotes. Moreover, they identified that oocyte-specific H3K27me3 was the DNA methylation-independent imprint. Interestingly, H3K27me3 imprinting was maintained during pre-implantation until the blastocyst stage. The ICM dilutes H3K27me3 imprinting and is completely lost in the epiblast at E6.5. However, at least *Gab1*, *Phf17*, *Sfmbt2*, *Slc38a4*, and *Smoc1* maintain H3K27me3 imprinting until E9.5 in the mouse placenta (Inoue et al., 2017a). Matoba et al. (2018) found that H3K27me3 imprinted genes exhibited biallelic expression and were completely dysregulated in mouse SCNT blastocysts. Furthermore, they found the absence of H3K27me3 imprinting in donor cells resulted in the loss of imprinted gene expression in the mouse SCNT embryos. Given that H3K27me3 imprinted genes are important for the development of the placenta (Itoh et al., 2000; Miri et al., 2013; Inoue et al., 2017b), loss of H3K27me3 imprinting may act as a barrier for post-implantation development in SCNT.

### Biallelic expression of *Xist* inhibits the development of somatic cell nuclear transfer embryos

In the female mammalian somatic cell, there are two X chromosomes (XX). One X chromosome and Y chromosome (XY) constitute sex chromosomes in males. To achieve X-linked dosage compensation between females and males, X-chromosome inactivation (XCI) has evolved (Sahakyan et al., 2018; Loda et al., 2022). In the XCI process, *Xist*, a non-coding RNA, is the trigger and master regulator of XCI (Penny et al., 1996; Engreitz et al., 2013; Loda and Heard, 2019). In mice, *Xist* first initiates at the paternally inherited X chromosome (Xp) and promotes imprinted XCI from the 4-cell to 8-cell stage in the female embryo. This type of XCI is called imprinted XCI. During pre-implantation, all cells maintain Xp imprinted XCI and a maternally inherited X chromosome (Xm) active state (Wang et al., 2016; Borensztein et al., 2017). Until the blastocyst stage, the trophectoderm still maintains imprinted XCI. However, the inner cell mass reactivates the paternal X chromosome and has two active X chromosomes. After implantation, random XCI, i.e., choosing Xp or Xm XCI at random, takes place in all cells of the embryo and is maintained in all descendent somatic cells throughout life (Lyon, 1962; Sahakyan et al., 2018).

In mouse cloned embryos, a previous study found that most X-linked genes are downregulated in SCNT pre-implantation embryos. Through immunofluorescent staining and RNA-FISH, they found ectopic expression of *Xist* in SCNT embryos, which caused two *Xist* puncta in female cells and a puncta in male cells; the result was both X chromosomes were inactivated (Inoue et al., 2010). Moreover, aberrant X chromosome inactivation patterns were reported in large animals (Xue et al., 2002; Jiang et al., 2008; Ruan et al., 2018). This result indicates that abnormal *Xist* expression is an epigenetic barrier for reprogramming in SCNT. It is worth mentioning that the XCI pattern is different among diverse mammals, and the abnormality of *Xist* expression may vary (Sahakyan et al., 2018; Patrat et al., 2020; Okamoto et al., 2021).

## Strategies to overcome epigenetic barriers

### Combination of various methods to achieve high cloning efficiency

To achieve high cloning efficiency, strategies need to be applied to correct the aforementioned epigenetic defects. Owing to diverse epigenetic barriers that exist in cloned embryos, the combination of these strategies has been used in many studies, and we can identify which combination worked well or was redundant (Table 1).

**TABLE 1:** Summary of cloned mammals after overcoming epigenetic barriers.

Species	Donor cell	Epigenetic modulations	No. of reconstructed embryo	No. of 2-cell (%)	No. of blastocyst (%)	No. of transferred embryo	No. of offspring (%)	References
Mouse	Cumulus	None	1,345	N/A	N/A	760	16 (2.1)	Wakayama et al. (1998)
Mouse	Sertoli	None	284	128 (45.1)	94 (33.1)	94	2 (2.1)	Ogura et al. (2000)
Mouse	Cumulus	TSA 100 nM/6 h	356	N/A	N/A	N/A	10 (2.8)	Rybouchkin et al. (2006)
Mouse	Cumulus	TSA 50 nM/10 h	178	170 (98)	N/A	170	11 (6.5)	Kishigami et al. (2006)
Mouse	Cumulus	Xist KO	239	225 (94.1)	N/A	100	12 (12)	Inoue et al. (2010)
Mouse	Sertoli	Xist KO	457	383 (83.8)	N/A	270	35 (13.0)	Inoue et al. (2010)
Mouse	Sertoli	*Xist* knockdown	125	89 (71)	N/A	89	11 (12)	Matoba et al. (2011)
Mouse	Sertoli	*Xist* knockdown + TSA 50 nM/10 h	85	69 (81)	N/A	69	14 (20)	Matoba et al. (2011)
Mouse	Cumulus	*Kdm4d*	76	92.7%	88.6%	119	9 (7.6)	Matoba et al. (2014)
Mouse	Sertoli	*Kdm4d*	102	89.3%	81.2%	92	8 (8.7)	Matoba et al. (2014)
Mouse	Cumulus	*Kdm4d* + Xist KO	N/A	N/A	N/A	75	14 (18.7)	Matoba et al. (2018)
Mouse	Sertoli	*Kdm4d* + Xist KO	N/A	N/A	N/A	85	20 (23.5)	Matoba et al. (2018)
Mouse	MEF	*Kdm4d* + Xist KO	N/A	N/A	N/A	29	2 (6.9)	Matoba et al. (2018)
Mouse	Cumulus	*Kdm4d* + *Kdm5b*	N/A	N/A	95%	N/A	11.1%	Liu et al. (2016)
Mouse	Cumulus	*Kdm4d* + *Kdm5b* Dnmt3a/3b siRNA	119	N/A	92.6%	63	11 (17.5)	Gao et al. (2018b)
Mouse	Cumulus	*Sfmbt2* miRNA KO	102	88 (86.3)	N/A	75	5 (6.7)	Inoue et al. (2020)
Mouse	TTF	Sfmbt2, Jade1, Gab1, Smoc1 monoallelic KO	135	121 (89.6)	28 (23)	49	7 (14.3)	Wang et al. (2020a)
Mouse	Cumulus	Dux + *Kdm4b*	N/A	N/A	N/A	20	5%	Yang et al. (2021)
Monkey	MEF	*Kdm4d* + TSA 10 nM/10 h	38	N/A	17 (44.7)	79	2 (2.5)	Liu et al. (2018b)
Cattle	Fibroblast	TSA 50 nM/10 h	237	222 (93.7)	103 (43.5)	36	3 (8.3)	Srirattana et al. (2012)
Pig	Fibroblast	Xist KO	332	N/A	121 (36.4)	530	11 (2.1)	Ruan et al. (2018)

N/A means not applicable. TSA 100 nM/6 h means 100 nM TSA treatment 6 hours. TSA 50 nM/10 h means 50 nM TSA treatment 10 hours.

### Overcoming H3K9me3 barrier promotes zygotic genome activation and pre-implantation development

In mice, *Matoba et al.* showed that overexpression of *Kdm4d*, an H3K9me3 demethylase, or injection of siRNA targeting *Suv39h1/2* H3K9me3 methyltransferases could repress the generation of H3K9me3 and improve the blastocyst rate to 87.5% in cumulus donors and the cloning efficiency up to 8.7% using Sertoli cells as the donor (Matoba et al., 2014). Liu et al. reported that overexpression of *Kdm4b*, another H3K9me3 demethylase, could also assist in overcoming the H3K9me3 barrier (Liu et al., 2016). The H3K9me3 barrier exists not only in mice but also in other large mammals. For example, *KDM4D* and *KDM4E* can rescue aberrant H3K9me3 deposition in bovine cloned embryos (Liu et al., 2018a). Injection of *KDM4D* or knockdown of *SUV39H1/H2* can improve the development efficiency in pigs (Weng et al., 2020; Liu et al., 2021). Moreover, two cloned monkeys were achieved successfully by injection of *KDM4D* in reconstructed embryos (Liu et al., 2018b; Liu et al., 2019). In the derivation of human ntESCs, overexpression of *KDM4A* can improve the human SCNT blastocyst rate and ntESC derivation efficiency (Chung et al., 2015). Interestingly, *Kdm4d* only rescued pre-implantation in ZGA of embryos because enlargement of the placenta was not solved after *Kdm4d* injection in mice (Matoba et al., 2014). This result indicates that the H3K9me3 barrier impacts ZGA and pre-implantation development.

### Removal of H3K4me3 barrier rescues 4-cell arrest in cloned embryos

In 2016, Liu et al. (2020) found that overexpression of *Kdm5b*, an H3K4me3 demethylase, rescued 4-cell arrest in mouse cloned embryos. Moreover, the combination of *K4m4b* and *Kdm5b* improved the SCNT blastocyst rate to over 95%, and 11.1% of reconstructed embryos developed to full term. Furthermore, in bovine cloned embryos, H3K4me3 in donor cells also was a barrier, and overexpression of *Kdm5b* improved the development of SCNT embryos.

### Overcoming H3K27me3 imprinting barriers improves post-implantation development of cloned embryos

In 2020, Wang et al. (2020a) reported that the H3K27me3 imprinting barrier could be overcome through monoallelic imprinting gene deletions of *Sfmbt2*, *Jade1*, *Gab1*, and *Smoc2* in fibroblasts, and these deletions increased fibroblast cloning efficiency to 14% compared with 0% in wild-type fibroblasts; these deletions also rescued the abnormal placental phenotype in SCNT. Moreover, monoallelic *Sfmbt2*, *Jade1*, and *Gab1* triple deletions or monoallelic *Sfmbt2* deletion also improved placental development and birth rate in SCNT. At the same time, Inoue *et al.* reported that correction of the expression of clustered miRNAs within the *Sfmbt2* gene could rescue SCNT abnormal placentas, and the combination of *Gab1* maternal deletion further alleviated abnormalities in SCNT placentas (Inoue et al., 2020). Recently, Xie et al. (2022) found that loss of *Slc38a4* imprinting was a significant cause of mouse placenta hyperplasia in SCNT embryos at late gestation. Indeed, these studies demonstrated that the H3K27me3 imprinting barrier affects cloned placenta and post-implantation development of SCNT.

### Histone deacetylase inhibitor improves pre-implantation development of cloned embryos

In 2006, two unrelated groups found trichostatin A (TSA), a histone deacetylase inhibitor, led to a more than 5-fold increase in birth rate and establishment of ntESCs successfully in mice (Kishigami et al., 2006; Rybouchkin et al., 2006). Since then, HDAC inhibitors have proven to increase *in vitro* development of SCNT embryos in other mammals, including sheep (Wen et al., 2014), cattle (Akagi et al., 2011; Min et al., 2016), pigs (Hou et al., 2014; Jin et al., 2017; Wang et al., 2018), gaurs (Srirattana et al., 2012), and monkeys (Liu et al., 2018b; Liu et al., 2019). However, the increase in birth rate has not been seen in these large animals aside from a study reported in pigs after scriptaid treatment (Zhao et al., 2010a; Ogura et al., 2021). Furthermore, the mechanism involved in the HDAC inhibitor’s improvement of cloning efficiency remains largely unknown. Recently, by using ULI-NChIP-seq (ultra-low-input native ChIP-seq), *Yang et al.* generated a high-resolution H3K9ac landscape of fertilized and SCNT embryos from the 1-cell to morula stage in mice and identified aberrantly acetylated regions (AARs) in SCNT embryos. Surprisingly, they found that TSA treatment was able to fix AARs without H3K9me3 occupancy and improve cloning efficiency. They also identified DUX, a crucial transcription factor that can correct aberrant H3K9ac signals in most AARs regardless of RRRs. Appropriate expression of exogenous *Dux* promotes *Dux* target genes and 2-cell expressed genes in mouse embryos. Consequently, it improves SCNT efficiency. Thus, in this study, TSA treatment worked in the aberrant H3K9ac regions of SCNT and rescued the efficiency of *Kdm4b*, and TSA largely relied on DUX (Yang et al., 2021). Prior to this result, Yang et al. (2020) found transient expression of *Dux* was able to facilitate reprogramming in cloned embryos.

### Fixing aberrant DNA methylation improves post-implantation development of somatic cell nuclear transfer embryos

In 2018, Gao et al. (2018b) found DNA re-methylation in pre-implantation SCNT embryos, and the knockdown of *Dnmt3a* and *Dnmt3b* improved the development of SCNT embryos, resulting in 5.33% of transferred embryos developing to term. More surprisingly, the increased weight of the SCNT placenta was alleviated after the knockdown of *Dnmt3a* and *Dnmt3b*. Through co-injection of *Kdm4b*+*5b* mRNA and *Dnmt3a*+*3b* siRNA, an impressive 17.2% of transferred embryos developed to term.

### Heterozygous knockout or knockdown of Xist improves cloning efficiency

In 2010, Inoue et al. (2010) reported that heterozygous knockout of *Xist*, which is overexpressed in cloned embryos, can improve the efficiency of SCNT by about 9-fold. Similarly, injection of *Xist* siRNA can improve female cloning efficiency by 10-fold compared to controls (Matoba et al., 2011). Moreover, the combination of *Xist* KO and *Kdm4d* overexpression can increase the development to full term of transferred SCNT embryos by 23% (Matoba et al., 2018).

### Modification of culture media improves the development of somatic cell nuclear transfer embryos

SCNT embryos are more sensitive to culture media than *in vivo* or *in vitro* fertilized embryos (Heindryckx et al., 2001; Chung et al., 2002; Dai et al., 2009; Cordova et al., 2017). Thus, much effort has been dedicated to improving the culture media for the development of SCNT embryos. In the mouse culture systems, KSOM, M16, and CZB are common mediums for fertilized embryos but cannot improve the development of SCNT using the canonical SCNT technology (Dai et al., 2009). Dai et al. (2009) reported that D media, a sequential culture method that involves M16 or CZB without EDTA and glutamine, used during early SCNT embryo development followed by transfer of the SCNT embryos to KSOM at the late 2-cell stage could significantly improve SCNT embryo development. Furthermore, 62.3% of SCNT embryos in D media culture can reach the blastocyst stage. Moreover, they found that deprivation of EDTA and glutamine from the D media overcame the arrest of the 2-cell stage in the development of SCNT, but the detailed mechanism responsible remains unknown. In 2002, Chung et al. (2002) found that supplementation of glucose in the CZB medium can improve early development in SCNT embryos but not in fertilized embryos. This result indicated that the metabolism and the requirements may differ between SCNT and fertilized embryos. Moreover, supplementation of melatonin or vitamin C, which is a free radical scavenger, in SCNT media can improve the blastocyst rate (Salehi et al., 2014; Mallol et al., 2015; Cordova et al., 2017). During the development of SCNT embryos, the metabolism and needs are diverse at different stages. Hence, transferring the SCNT embryos into the oviduct at the 2-cell stage is preferable (Cordova et al., 2017). The two-step cultured medium is also suitable for other mammals in the development of SCNT, including cattle, goats, sheep, and rabbits (Wang et al., 2011; Kwong et al., 2012; Hosseini et al., 2015; Sugimoto et al., 2015).

## Conclusion

Somatic cell nuclear transfer technology was developed more than 60 years ago, and the first cloned mammal, Dolly, was generated over 25 years ago. Many breakthroughs have been made, especially recently. Following the development of low-input sequencing technology, we can analyze epigenetic changes in SCNT embryos and explore the mechanisms of SCNT reprogramming.

In this review, we describe the recently discovered epigenetic barriers and discuss the strategies to overcome these barriers. From the aforementioned studies, most barriers in SCNT embryos, including pre-and post-implantation barriers, could be overcome using multiple methods. Moreover, a combination of different and non-redundant methods has the potential to achieve high cloning efficiency.
